# Empathy During Crises: Investigating Attitudes, Tolerance, and Ingroup–Outgroup Dynamics in Response to Refugee Movements

**DOI:** 10.1111/jopy.13012

**Published:** 2025-01-27

**Authors:** Ronja Demel, Lena Masch, David Schieferdecker, Hanna Schwander, Swen Hutter, Jule Specht

**Affiliations:** ^1^ Humboldt‐Universität Zu Berlin Berlin Germany; ^2^ Freie Universität Berlin Berlin Germany

**Keywords:** closeness, empathy, intergroup relations, refugees, tolerance

## Abstract

**Objective:**

In times of societal crises, it is pivotal to understand and share others' feelings. Yet, the role of empathy in fostering prosocial responses during societal crises has not gained enough attention. Our study uses the onset of Russia's war on Ukraine to examine three key questions: (1) Is empathy related to attitudes toward pro‐refugee policies? (2) Does empathy correlate with a higher tolerance for diverse opinions on refugee policies? (3) Is empathy linked to perceived interpersonal closeness toward social in‐ and outgroups, including refugees?

**Methods and Results:**

Using observational data from online surveys conducted with a largely representative quota sample from Germany (*N* = 1199–1631) during the initial months of the war, we found that empathy was associated with significantly higher support of pro‐refugee policies, driven primarily by empathic concern. Additionally, more empathetic individuals exhibited greater opinion diversity and showed smaller distinctions in perceived interpersonal closeness between in‐ and outgroups.

**Conclusions:**

These findings underscore the critical role of empathy in fostering solidarity and social cohesion during societal crises.

## 1 Introduction

Empathy describes the ability to understand and feel with another person's emotions (Batson 1990). Feeling with someone and understanding that people have different perspectives on life form the foundation for solidarity and social cohesion. These processes become particularly crucial during societal crises when resource conflicts and uncertainty threaten to erode the social fabric (e.g., Rosler, Cohen‐Chen, and Halperin 2017).

Russia's war on Ukraine has caused a significant political crisis in Europe. While military operations have been largely confined to Ukraine's physical territory and primarily affect the Ukrainian population, the war has had substantial repercussions for other European countries. Societies are facing questions about their vulnerability to military and hybrid attacks in light of potentially failing NATO deterrence strategy (Magula, Rouland, and Zwack 2022). Governments have had to reassess their plans to transform the energy sector, balancing the reduction of coal consumption and the phasing out of nuclear energy with a heavy dependence on Russian natural gas (Aitken and Ersoy 2023; Gajdzik et al. 2024). Moreover, the war had led to the largest displacement of people in Europe since World War II (UNHCR 2023a).

In this study, we focus on the humanitarian aspect of the war. To better understand the role of empathy in fostering solidarity and social cohesion during societal turmoil, we examined the role of empathetic concern and perspective‐taking on attitudes toward pro‐refugee policies, tolerance toward opinion diversity on these policies, and feelings of interpersonal closeness toward social in‐ and outgroups. To this end, we analyzed survey data collected from a largely representative sample of the German population that we collected in the first weeks and months following Russia's invasion. Germany provides a suitable case in point where heated debates have arisen regarding foreign policy and the distribution of resources after a surplus budget of 100 billion EUR was set aside for military expenditures (Mello 2024), inflation surged to 7.9%, and over a million Ukrainian refugees arrived in the country in 2022 (Statistisches Bundesamt 2023).

### 1.1 Empathy and Its Facets

Empathy is defined as the ability to understand another person's emotions and motivations (Batson 1990). Empathy encompasses both a state and a trait. State empathy refers to the response of feeling with someone in a specific situation. Trait empathy, on the other hand, represents a more enduring and general capacity to feel with others (Van der Graaff et al. 2016). Despite being more permanent, trait empathy can evolve over the course of a life and through training interventions (for a meta‐analysis, see e.g., Teding van Berkhout and Malouff 2016).

In his authoritative review, De Waal (2008) distinguished three facets of empathy: emotional contagion, sympathetic concern, and empathetic perspective‐taking. Emotional contagion involves the reflex‐like adoption of another person's emotional state, reacting to perceived distress with one's own distress. Sympathetic (or empathetic) concern extends beyond emotional contagion in that individuals also appraise the causes and contexts of the other person's emotions. While sympathetic concern does not necessarily involve the sharing of emotions, it has been demonstrated to promote helping behavior (Batson et al. 2007; Eisenberg, Eggum, and Di Giunta 2010). Finally, empathetic perspective‐taking refers to the cognitive understanding of another person's distinct situation, enabling the recognition of their emotions without necessarily experiencing them oneself (De Waal 2008; Eisenberg et al. 2006).

This study focuses on trait empathy and its two facets of empathic concern and perspective‐taking as the two other‐oriented facets of empathetic responses. The two facets can influence how we perceive others and, consequently, our behavioral intentions to support individuals in need (Batson et al. 2007). They can be seen as manifestations of “other‐oriented, altruistic motivation” (Eisenberg 2000, 667). Perspective‐taking emphasizes the cognitive ability to comprehend the internal states and external circumstances of others in need, even if they differ from one's own situation (Eisenberg et al. 2006). In contrast, empathic concern highlights the emotional capacity to value another person's well‐being. The relevance of these capacities varies across situations, depending on which mechanism—cognitive understanding or emotional response—is activated and required.

### 1.2 Empathy and Attitudes on Pro‐Prosocial Policy

This study seeks to understand the role of empathy in shaping public response to the Russian war on Ukraine and the arrival of millions of Ukrainians in Germany. Prior research indicates that empathy can foster voluntary contributions to public goods and deter individuals from harming others for personal gain (for a review, Davis 2015). Additionally, studies suggest that individuals with higher levels of empathy tend to show greater support for minority groups (Politi et al. 2023; Taylor and McKeown 2021; Zheng et al. 2021). Ultimately, empathy can diminish the reliance on institutional enforcement mechanisms (Ostrom 2000).

Applied to the context of the war, empathy may facilitate solidarity with those fleeing Ukraine—for example, in the form of donating goods and money, providing housing, or supporting pro‐refugee policies. Initial evidence indicates that segments of the civil society reacted to the arrival of Ukrainian refugees with offers of assistance (Höltmann, Hutter, and Rößler‐Prokhorenko 2022), and potentially, this solidarity has been catalyzed by the high empathy of those who took action. However, there are reasons to be skeptical that the relationship between empathy and solidarity materializes in the specific context of refugee movements.

The topic of refugees and the politics surrounding refugee movements remain highly contentious in Germany and many other recipient countries (see Esses 2021, for a comprehensive review). The issue has been particularly divisive since the significant increase in refugee numbers in 2015, leading to polarization within European countries on refugee‐related matters (e.g., Albada, Hansen, and Otten 2021; De Coninck 2020; Helbling, Maxwell, and Traunmüller 2023). Higher aversion toward refugees has often been linked to conservative political orientations (Plener et al. 2017) and heightened perceptions of threat (Igarashi 2021). In particular, supporters of far‐right parties advocate against immigration and are less likely to endorse or assist migrants and refugees (Mudde 2019). Relatedly, framing refugees as “illegitimate immigrants” (De Coninck 2020) can exacerbate anti‐migration sentiments (Hutter and Kriesi 2022). Consequently, the issue of refugees and migration has led to substantial polarization, with distinct pro‐ and anti‐refugee factions (Albada, Hansen, and Otten 2021; Lönnqvist, Ilmarinen, and Sortheix 2020). This is particularly pronounced in Western European societies where polarization between left‐wing and right‐wing parties is on the rise (van der Brug and Harteveld 2021).

### 1.3 Trait Empathy and the Tolerance for Diversity of Political Opinions

In the context of highly polarized issues like refugee movements, empathy may not only foster solidarity toward refugees but may also promote social cohesion within society. In other words, empathy may not only be related to a prosocial stance toward those in need but also to a heightened ability to understand and tolerate that people form different opinions on the issue. Previous research indicates that individuals with higher empathy tend to exhibit greater tolerance toward opposing viewpoints (Butrus and Witenberg 2013; Hu and Lee 2018).

Having said this, it remains an empirical question whether a similar relationship exists for contentious issues such as pro‐refugee policies. For supporters of such policies, accepting diversity of opinions can conflict with their empathy and solidarity toward refugees. Moreover, the strength of an opinion may matter: Research suggests that moderate views tend to correlate with greater tolerance, whereas more extreme views have been associated with less tolerance toward opposing perspectives (Ganzach and Schul 2021; Toner et al. 2013). When divergent opinions clash with deeply held beliefs, individuals with more extreme views may exhibit greater rigidity and difficulty engaging in cognitive complexity and perspective‐taking (Jugl 2022). Lastly, it is plausible that mainly the cognitive facet of empathy—perspective‐taking—influences tolerance toward differing views of refugee‐related policies.

### 1.4 Trait Empathy and Interpersonal Closeness

Empathy may be important for social cohesion beyond mere tolerance for diverse opinions. Research has consistently shown that individuals tend to exhibit greater empathy toward those who share similarities with them (Eklund, Andersson‐Stråberg, and Hansen 2009; Stürmer et al. 2006; Stürmer and Siem 2017) and less empathy toward outgroup members—those perceived as different in at least one salient characteristic (Arceneaux 2017; Borinca, Falomir‐Pichastor, and Andrighetto 2020; Gutsell and Inzlicht 2012; Lotz‐Schmitt, Siem, and Stürmer 2017; Tarrant, Dazeley, and Cottom 2009). This phenomenon has been observed across various traits such as race or ethnicity (e.g., Arceneaux 2017) and even in minimal group settings where individuals are randomly assigned to groups based on arbitrary characteristics (e.g., Masten, Gillen‐O'Neel, and Brown 2010; Montalan et al. 2012). Moreover, in competitive settings, this ingroup bias in empathy can transform into counter‐empathy, where individuals may experience pleasure at others' pain and pain at others' pleasure (Cikara, Bruneau, and Saxe 2011, for a review; Cikara et al. 2014). However, empathy can also help to bridge ingroup–outgroup divides (Barth and Stürmer 2016; Batson et al. 2002; Bruneau and Saxe 2012; Malhotra and Liyanage 2005; Todd, Bodenhausen, and Galinsky 2012). Studies have demonstrated that empathy inductions can significantly impact perceptions and attitudes toward stigmatized outgroups (Batson et al. 2002), foster greater understanding and solidarity with marginalized outgroups in intergroup conflicts (Barth and Stürmer 2016; Bruneau and Saxe 2012; Malhotra and Liyanage 2005), and raise awareness of intergroup discrimination while reducing prejudice (Alan et al. 2021; Bobba and Crocetti 2022; Todd, Bodenhausen, and Galinsky 2012).

Thus far, the existing literature indicates that empathetic responses vary when directed toward outgroup members, but once empathic responses are elicited, they can positively influence attitudes toward those outgroups (e.g., Barth and Stürmer 2016; Cikara, Bruneau, and Saxe 2011; Malhotra and Liyanage 2005). Having said this, there has been a notable lack of studies focusing on responses to refugees (e.g., Taylor and McKeown 2021). Given the rising numbers of refugees around the world (UNHCR 2023b), this is an oversight of a phenomenon of utmost social relevance. Moreover, studying empathy in the context of refugee movements is promising for theoretical reasons: Refugees are often perceived as outgroup members by citizens of the receiving country not only because of their foreign birthplace but also due to (real or perceived) differences in ethnicity, racialized group, religion, and/or language. At the same time, many refugees have endured significant humanitarian hardships that should make an empathetic response more likely. Moreover, responses to different groups of refugees may vary; some refugee outgroups could be perceived as psychologically more distant if they differ along multiple aforementioned dimensions (see also Trope and Liberman 2010).

The context of the Russian war on Ukraine provides a valuable case for examining how empathy affects the differential reactions to refugees in the domestic population of a recipient country. Alongside Ukrainian refugees, large numbers of people from Syria—as well as other countries in the Middle East and North Africa—have sought refuge in Germany in recent years (BMI 2022). It is plausible to assume that people born in Germany may perceive more psychological distance from refugees originating from Syria than Ukrainian refugees. This perception could stem from geographic distance and/or the historical dominance of different religions. Furthermore, people from the Middle East and Northern Africa are more likely to be racialized and portrayed in public discourse as threatening (e.g., Berning and Schlueter 2016; De Coninck 2022; Meidert and Rapp 2019), potentially exacerbating perceptions of them as ‘distant others’. In line with this reasoning, first research suggests that refugees have not been equally welcomed by citizens in Germany (and other European countries; Moise, Dennison, and Kriesi 2024): While Ukrainian refugees have generally been met with significant solidarity by the civil society in Germany (Höltmann, Hutter, and Rößler‐Prokhorenko 2022), refugees from Middle Eastern and Northern African countries have encountered substantial pushbacks at the European borders (e.g., Tondo 2022), less welcoming attitudes from the public (Dollmann et al. 2022), and less support from civil society (see e.g., Letki et al. 2022) and public policies (e.g., in the form of the recognition of educational diplomas, processing of residence status, or access to work permits; see e.g., Prange 2022). Therefore, our interest extends beyond examining whether higher empathy correlates with a more sympathetic response to refugees. We also seek to understand whether empathy can mitigate differences in responses toward different refugee groups.

### 1.5 Research Questions and Hypotheses

The current study examines the role of trait empathy in shaping perceptions and attitudes in the context of the refuge of millions of Ukrainian citizens in Germany, exploring three interconnected research questions. Our first research question investigates whether higher empathy correlates with more positive attitudes toward pro‐refugee policies (RQ1). Drawing from our literature review, we hypothesize that empathy is associated with more support of policies regarding the unconditional admission of refugees, access to social benefits for refugees, and the issuing of work permits (H1a). Here, we anticipate that these associations will be primarily driven by empathic concern (H1b).

In our second research question, we investigate whether empathy is linked to a greater understanding of diverse perspectives regarding the highly polarized issue of pro‐refugee policies (RQ2). Based on previous research, we tentatively expect that more empathetic individuals will exhibit increased tolerance for diverse opinions (H2a). We further posit that this relationship will be particularly influenced by higher levels of perspective‐taking (H2b). Moreover, we expect that the extremity of an individual's own opinion matters: those with more extreme views will display lower tolerance for opinion diversity, whereas those with moderate stances will show greater tolerance for differing opinions (suggesting a curvilinear relationship; H2c).

Our third research question asks whether empathy is associated with perceptions of closeness toward others (RQ3). We hypothesize that individuals with higher levels of empathy will report feeling greater interpersonal closeness toward others compared to those with lower empathy (H3a). Additionally, we expect that levels of interpersonal closeness will vary based on ingroup/outgroup categorization of others. Specifically, in the context of Germany, we expect that individuals will perceive their national ingroup (Germans) as closest and refugees as closer when they belong to a national group perceived as less psychologically distant (Ukrainians) rather than more distant (Syrians; H3b). Having said this, we predict that empathy will be associated with reduced group bias: more empathic individuals will differentiate less between nationalities (H3c). Finally, we expect that these relationships will be primarily driven by the facet of empathic concern (H3d).

## 2 Materials and Methods

### 2.1 Study Design and Implementation

To answer our research questions, we utilize original data from a large interdisciplinary online survey study conducted in Germany. The longitudinal panel survey aims to document shifts in public opinions within the German population since the onset of the Russian war in Ukraine. The survey was initiated just days after the Russian invasion in February 2022. The data, along with the code to replicate our analyses and a codebook with the survey questions, are available at DOI: 10.17605/OSF.IO/E5XUZ.

This study uses the data from various survey waves in a cross‐sectional design, as we did not anticipate systematic changes in trait empathy within the sample or in the relationship between trait empathy and the outcomes under study during the initial months of the war. Specifically, we included data from the first six waves of the online panel, with data collections occurring between March 8 and September 22, 2022, at intervals of 2–6 weeks. The sample was recruited through the online panel provider Bilendi, and the surveys were administered via the Unipark online survey tool. Each panel wave took approximately 5 min to complete, and participants were incentivized with 0.25–0.40€ (depending on the medium length of the respective survey), plus an additional 2€ for completing a set of four waves. The surveys fully disclosed the aims of the study. Participants were informed about the risks, compensation, and their rights and provided written consent. After completing each survey wave, participants were debriefed and received their incentive from the panel provider. Due to the ad‐hoc nature of the initial data collection, the study design and analysis were not preregistered. Data collection was conducted in accordance with the Declaration of Helsinki (World Medical Association 2001) and was granted ethical clearance by the Institute of Psychology at Humboldt University of Berlin (Date: 02 September 2022).^1^

### 2.2 Participants

The targeted sample size for the panel study was 2000 complete cases in Wave 1 and, accounting for panel attrition, at least 1000 complete cases in subsequent waves. To ensure a largely representative sample of the German population, quotas were set in wave 1 for age (18–69 years), gender (female, male),^2^ education (low = no qualified schooling (yet), up to 10 years of secondary school with or without vocational training; medium = completed secondary school without general qualification for university entrance or a comparable degree; high = completed secondary school with general qualification for university entrance or higher), and state of residence (covering all 16 German states).

Participants were excluded from the panel if they did not meet the age range of 18–69 years (*n* = 5) or if they completed the first survey wave in less than 2 min (*n* = 661). Due to the low case numbers, individuals who did not identify with the binary gender categories that the panel provider predefined were also excluded (*n* = 6). Additionally, participants with technical difficulties, such as duplicate IDs or missing duration data, were excluded (*n* = 11). For our main analyses, we only considered participants who provided information on all four items of our empathy measure (see below). To investigate RQ1 and RQ2, we included participants who also responded to at least one attitude and one understanding item for each of the three policy items across all six waves (*n* = 1631). For our analysis of RQ3, we only included participants who, in addition to meeting the aforementioned criteria, answered all six “Inclusion of Other in the Self” items (*n* = 1199). Table 1 describes both samples in terms of their socio‐demographic characteristics.

**TABLE 1:** Descriptive statistics of socio‐demographic factors, empathy, and tolerance for opinion diversity for the two subsamples.

	Sample RQ1 and 2	Sample RQ3
Age	47.09 years (13.87 years)	48.80 years (13.37 years)
*Gender*		
Male	51%	54%
Female	49%	46%
*Education*		
Low	28%	27%
Medium	34%	34%
High	39%	39%
*Region*		
East	14%	15%
West	81%	80%
Berlin	5%	6%
*Born in Germany*		
Yes	96%	96%
No	4%	4%
*Parents born outside Germany*		
Yes	17%	16%
No	83%	84%
Don't know/no answer	0%	0%
*Empathy*		
Empathic concern	3.58 (0.77)	3.56 (0.76)
Perspective‐taking	3.20 (0.78)	3.18 (0.77)
*Tolerance toward opinion diversity*		
Admission	10.82 (2.19)	—
Social benefits	10.95 (2.05)	—
Work permits	10.88 (2.15)	—
*N*	1631	1199

*Note:* Cells display mean (SD) for metric variables. Percentages for categorical variables are rounded. Tolerance toward opinion diversity is not part of the analysis of the RQ3 sample and is thus not reported.

### 2.3 Measurement

We measured *trait empathy* and the two subdimensions, *empathic concern* and *perspective‐taking*, in wave 2. Participants completed the short version of the Saarbrücker Persönlichkeitsfragebogen (Paulus 2016), which is a German version of the Interpersonal Reactivity Index (IRI; Davis 1980). Empathy was assessed using four items: two items assessed empathic concern (“I have warm feelings for individuals who are less well off than I am.” and “Things affect me very much, even if I only observe them.”), and two items assessed perspective‐taking (“When someone else's behavior seems strange to me, I try to put myself in their situation for a while.” and “Before I criticize someone, I try to imagine how I would feel if I were in their position.”). Respondents were given a 5‐point scale to answer (1 = *never*, 5 = *always*). In our analyses, overall empathy was defined as the mean score of all four items; empathic concern and perspective‐taking were defined as the mean score of their respective items, respectively. Cronbach's alphas suggested an acceptable to good inter‐item‐reliability (empathy *α*_s_ = 0.79; empathic concern *α*_s_ = 0.79; perspective‐taking *α*_s_ = 0.73).

Participants were asked about their *attitudes toward pro‐refugee policies* in each of the six waves. Specifically, we asked for their agreement with three items that covered the domains of refugee admission (“Germany should take in as many refugees from Ukraine as possible, even when other European countries take in fewer refugees”); access to social benefits (“Refugees from Ukraine should be able to receive social benefits in Germany”); and work permits (“Refugees from Ukraine should be allowed to work in Germany”). All three items were rated on an 11‐point scale (0 = *strongly disagree*; 10 = *strongly agree*). Cronbach's alpha for the three items was *α* = 0.86, indicating a good semantic overlap between them.

We measured *tolerance for opinion diversity* in each of the six waves. Right after each item that measured attitudes toward a pro‐refugee policy, participants were asked to report their understanding of people with differing opinions on the policy in two items. Specifically, we asked “How much understanding do you have for people who are in favor of [*policy*]?” and then repeated the item asking for “people who are against [*policy*]”. Answering options ranged from “0 = *no understanding*” to “10 = *full understanding*”. For subsequent analyses, we computed a mean score per participant across all survey waves for each of the two items for each policy attitude. Tolerance toward opinion diversity was defined as the sum of the understanding of the pro and con attitudes of each political stance for each participant and across all waves (e.g., the sum score of understanding toward individuals who are in favor of refugee admission and understanding toward individuals who are against refugee admission).

**FIGURE 1:**
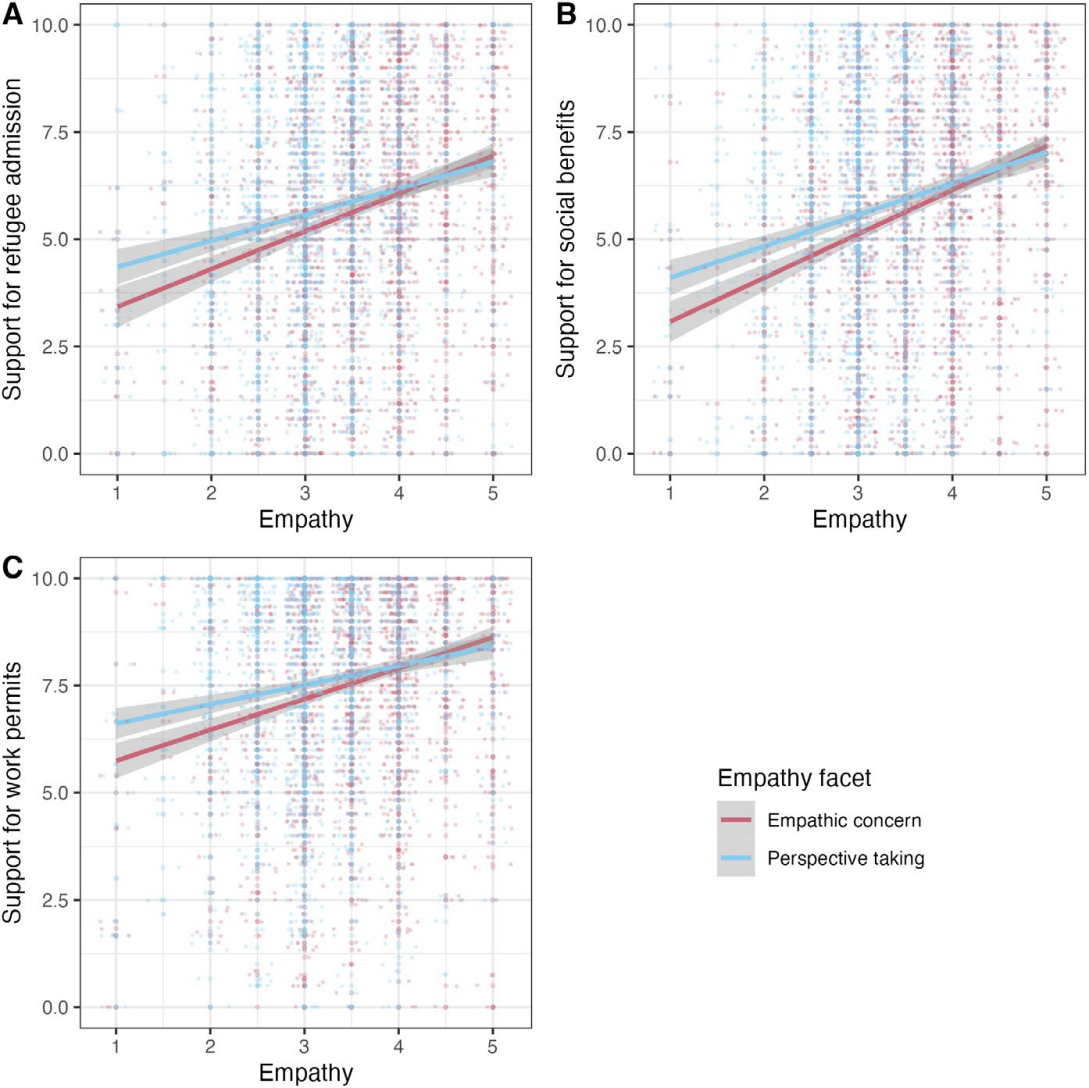
H1b: Relationship between empathic concern, perspective‐taking, and attitudes toward pro‐refugee policies. Regression lines for both facets of empathy, empathic concern (red), and perspective‐taking (blue) on (A) support for refugee admission, (B) social benefits, and (C) work permits. Gray‐shaded areas indicate 95% confidence intervals.

To measure *interpersonal closeness*, we presented participants with a variation of the “Inclusion of Other in the Self (IOS)”‐scale (Aron, Aron, and Smollan 1992) in wave 6. In six questions, participants were asked to rate how close they felt toward another person. The other person was only described in terms of their nationality/refugee status and gender. The six persons included a “German woman,” a “German man,” a “fled Ukrainian woman,” a “fled Ukrainian man,” a “fled Syrian woman,” and a “fled Syrian man.” The IOS‐answering scale consists of seven pairs of circles (see Figure 1). In each pair, one circle represents the “self” and one circle the “other”. The amount of overlap between the circles varies and is considered an indicator of the degree of perceived closeness (with higher overlaps indicating higher closeness). Using sliders, participants could indicate how close they felt to the other person. (Compared to the original IOS scale, we slightly increased the distance between the circles as we aimed to capture the relationship between strangers, whereas the original scale was developed in the context of romantic relationships.).

We measured various *socio‐demographics* in wave 1. Participants answered socio‐demographic questions including their age (in years); gender (male, female, non‐binary); state of residence (all 16 German states); highest education level (low, medium, high; see above); whether they were born in Germany (yes, no); and whether any of their parents were not born in Germany (yes, no, do not know). States of residence were aggregated to represent the historical East (without Berlin), West (without Berlin), and Berlin (the Eastern and Western parts of the city).

### 2.4 Statistical Analyses

Data were analyzed in R (R Core Team 2022, version 4.2.3) using R Studio. Standard *p*‐values of 0.05 were used as a cut‐off for two‐tailed distributions. All continuous predictors and control variables were *z*‐transformed before being included in the models. The gender and age of participants were included in all models as control variables. The gender of the “other” person was included as a control variable in the model for RQ3. Tables were produced via the function ‘tab_model’ (R package “SjPlot”, Lüdecke 2022), and graphs were created using ‘ggplot’ (Wickham et al. 2022).

#### 2.4.1 Analyses for RQ1

To understand how empathy is related to attitudes toward pro‐refugee policies (H1a), we ran a linear regression with empathy and the aforementioned control variables as predictors and the mean across all refugee items as the outcome. We fitted the fixed‐effects‐model using the function ‘lm’ and checked various diagnostics of model validity and stability (Cook's distance, DFBetas, DFFits, leverage, and variance inflation factors; distribution of residuals, residuals plotted against fitted values) using the functions ‘vif’ (R package “car”, Fox, Weisberg, and Price 2022), ‘dffits’, ‘dfbeta’, ‘cooks. distance’, and ‘qqplots’. None of these checks indicated highly influential cases, nor obvious deviations from the assumptions of normality and homogeneity of residuals.

To understand how the facets of empathy are related to each of the refugee items individually (H1b), we ran a multiple linear regression for each of the three refugee items separately with empathic concern, perspective‐taking, and the control variables as predictors. Analyzing the three items separately allowed us to make more precise statements about agreement with the individual policies and the influence of empathy on them. We similarly fitted the model as for (H1a) and observed no obvious deviations from model assumptions.

#### 2.4.2 Analyses for RQ2

To test whether empathy is related to tolerance toward opinion diversity (H2a), we computed a linear regression with empathy and the aforementioned controls as predictors and the mean across all refugee items as an outcome. We fitted the model in the same way as in H1a and ran the same diagnostics. ‘qqPlots’ indicated slight deviations from normality due to a heavy‐tailed error distribution; however, we decided that the linear regression is suitable since (a) the deviations were small, (b) estimators in linear regression models are not particularly sensitive to these deviations, and (c) all other assumptions were fulfilled.

To understand whether empathic concern and perspective‐taking are related to tolerance toward opinion diversity in different ways (H2b), we ran a multiple linear regression for each of the three policies, using empathic concern, perspective‐taking, and the control variables as predictors and tolerance toward opinion diversity on the respective policy as the outcome. We fitted the model in the same way as in H1a and ran the same diagnostics. Again, diagnostics suggested the existence of potentially influential cases, yet a robustness check excluding these cases revealed no substantial differences in results.

To test whether tolerance toward opinion diversity on a policy issue is related to respondents' attitude (strength) regarding the respective issue (H2c), we ran a multiple regression for each of the three policies in which we included participants' attitude regarding the policy as a linear, quadratic, and cubic effect. As such, we could test for a non‐linear, asymmetric relationship. We fitted the model in the same way as in H1a and ran the same diagnostics. Dfbetas and leverage values indicated the existence of potentially influential cases; however, a robustness check in which we re‐ran each model excluding values above the leverage threshold did not reveal substantial differences in effect direction and patterns of significance (see Table S1).

#### 2.4.3 Analyses for RQ3

To examine RQ3, we restructured the data so that the ratings for each scenario described in the IOS circles were split by nationality and gender. This resulted in a repeated measures design with six observations per person (functions “gather” and “separate” of the R package “tidyr”, Wickham and Girlich 2022). The sample of each of the models for these analyses was a total of 7194 observations made on 1199 individuals.

To test whether more empathetic people feel closer to others compared to individuals with less empathy (H3a), we used a linear mixed model (Baayen, Davidson, and Bates 2008) in which we included interpersonal closeness as an outcome, empathy as a predictor, and national group and gender of the “other” as random effects. The model was fitted using the function ‘lmer’ of the R package lme4 (Bates et al. 2022).

To test whether interpersonal closeness is related to the nationality of the “other” (H3b) and whether biases in interpersonal closeness based on nationality differed for individuals with higher compared to lower empathy (H3c), we fitted the same model as for H3a but added the nationality of the “other” as well as its interaction with empathy as predictors and the main effect of the gender of the “other” as a control variable.

To understand whether empathic concern and perspective‐taking are differently related to interpersonal closeness (H3d), we repeated the analyses of H3b but included empathic concern and perspective‐taking separately in the model as well as their interactions with nationality.

For all models, we ran diagnostics and found no indications of a violation of the assumptions of normally distributed and homogenous residuals, the existence of influential cases, or collinearity. No insubjects exist. Variance inflation factors were derived using the ‘vif’ function (Fox, Weisberg, and Price 2022) applied to a standard linear model excluding the interactions and random effects and did not indicate collinearity to be an issue.

## 3 Results

### 3.1 Empathy and Attitudes Toward Pro‐Refugee Policies (RQ1)

Inspecting the univariate distributions, our data indicates that the participants were, on average, supportive of pro‐refugee policies, particularly for the granting of work permits (*M*_admission_ = 5.70, SD_admission_ = 2.86; *M*_benefits_ = 5.72, SD_benefits_ = 2.90, *M*_work_ = 7.60, and SD_work_ = 2.47). Having said this, the relatively large standard variations suggest a considerable variance in attitudes.

Supporting H1a, the results of our multivariate analysis indicate that individuals with more empathy showed more positive attitudes toward pro‐refugee policies controlling for various socio‐demographics (*β* = 0.70, *p <* 0.001, CI [0.58–0.81]; full model in Table S2).

**FIGURE 2:**
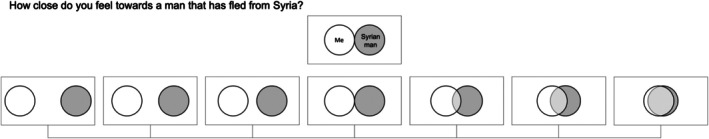
Illustration of the measurement of interpersonal closeness. One example of interpersonal closeness ratings, adapted from Aron, Aron, and Smollan (1992). The two pairs of circles represent the self and the other person and vary in their amount of overlap as an indicator of closeness toward the other person. The other person was varied regarding their national group (German, Ukrainian, Syrian) and gender (male, female).

Supporting H1b, we found that the association between empathy and support of pro‐refugee policies was mainly driven by the facet of empathetic concern. Higher empathic concern was associated with significantly more positive attitudes toward refugee admission (Figure 2A), social benefits (Figure 2B), and work permits (Figure 2C). In contrast, higher perspective‐taking was only associated with a significantly stronger endorsement of social benefits (Table 2).

**TABLE 2:** H1b: The relationship of empathic concern and perspective‐taking with attitudes regarding refugee admission, social benefits, and work permits.

Predictors	Refugee admission				
	Estimate	CI	SE	*z*	*p*
(Intercept)	5.28	5.09 to 5.47	0.10	53.55	**< 0.001**
Empathic concern^a^	0.68	0.52 to 0.84	0.08	8.30	**< 0.001**
Perspective‐taking^a^	0.14	−0.02 to 0.30	0.08	1.70	0.090
Gender [male]^b^	0.83	0.55 to 1.10	0.14	5.88	**< 0.001**
Age^a^	−0.12	−0.25 to 0.02	0.07	−1.68	0.094
Observations	1631				
*R* ^2^/*R* ^2^ adjusted	0.078/0.075				

Predictors	Social benefits				
	Estimate	CI	SE	*z*	*p*
(Intercept)	5.36	5.17 to 5.56	0.10	54.16	**< 0.001**
Empathic concern^a^	0.74	0.58 to 0.90	0.08	8.95	**< 0.001**
Perspective‐taking^a^	0.20	0.04 to 0.36	0.08	2.51	**0.012**
Gender [male]^b^	0.70	0.42 to 0.98	0.14	4.95	**< 0.001**
Age^a^	−0.02	−0.16 to 0.11	0.07	−0.34	0.736
Observations	1631				
*R* ^2^/*R* ^2^ adjusted	0.091/0.088				

Predictors	Work permit				
	Estimate	CI	SE	*z*	*p*
(Intercept)	7.30	7.13 to 7.46	0.09	85.28	**< 0.001**
Empathic concern^a^	0.56	0.42 to 0.70	0.07	7.80	**< 0.001**
Perspective‐taking^a^	0.09	−0.05 to 0.23	0.07	1.25	0.210
Gender [male]^b^	0.60	0.37 to 0.84	0.12	4.95	**< 0.001**
Age^a^	0.11	−0.01 to 0.23	0.06	1.85	0.064
Observations	1631				
*R* ^2^/*R* ^2^ adjusted	0.069/0.066				

*Note:* Coefficients of a linear regression of empathy concern and perspective‐taking on attitudes regarding refugee admission (top panel), access to social benefits (middle panel), and grating work permits (bottom panel), with gender and age as control variables. Estimates are standardized coefficients.

^a^ *z*‐transformed.

^b^ With female as reference category.

### 3.2 Empathy and Tolerance Toward Opinion Diversity (RQ2)

Inspecting the univariate distribution, our data suggests that participants on average scored in the middle of our scale when it comes to tolerance toward opinion diversity regarding all three pro‐refugee policies (*M*_admission_ = 10.82, SD_admission_ = 2.19; *M*_benefits_ = 10.88, SD_benefits_ = 2.15; *M*_work_ = 10.95, SD_work_ = 2.05; a theoretical range of 0–20). Importantly, this does not mean that participants had “medium levels” of tolerance; they rather showed “baseline levels” of tolerance in which they either had (a) some understanding of both positions or (b) a high understanding of one position and little understanding of the other.

Supporting H2a, we found that higher empathy was associated with higher tolerance toward opinion diversity, controlling for various socio‐demographics (*β* = 0.95, *p* < 0.001, CI [0.66–1.23]; full model in Table S3).

When analyzing the relationships between the facets of empathy and individual policies, we found tentative support for H2b (Table 3). Higher perspective‐taking was significantly associated with more tolerance toward opinion diversity regarding refugee admission, whereas empathic concern showed no significant relationship with tolerance. Having said this, perspective‐taking had no significant relationship to support of social benefits or work permits for refugees.

**TABLE 3:** H2b/H2c: The relationship of empathic concern, perspective‐taking, one's own opinion, and tolerance for opinion diversity regarding refugee admission, social benefits, and work permits.

Predictors	Refugee admission				
	Estimate	CI	SE	*z*	*p*
(Intercept)	10.79	10.61 to 10.96	0.09	120.33	**< 0.001**
Empathic concern^a^	0.08	−0.04 to 0.21	0.06	1.30	0.192
Perspective‐taking^a^	0.13	0.01 to 0.25	0.06	2.16	**0.031**
Attitude admission^a^	1.12	0.88 to 1.36	0.12	9.07	**< 0.001**
(Attitude admission)^2^ ^a^	−0.07	−0.19 to 0.05	0.06	−1.17	0.243
(Attitude admission)^3^ ^a^	−0.24	−0.35 to −0.13	0.06	−4.27	**< 0.001**
Gender [male]^b^	0.05	−0.16 to 0.26	0.11	0.47	0.641
Age^a^	−0.18	−0.28 to −0.08	0.05	−3.49	**0.001**
Observations	1631				
*R* ^2^/*R* ^2^ adjusted	0.120/0.116				

Predictors	Social benefits				
	Estimate	CI	SE	*z*	*p*
(Intercept)	10.96	10.79 to 11.13	0.09	125.39	**< 0.001**
Empathic concern^a^	0.07	−0.05 to 0.19	0.06	1.20	0.232
Perspective‐taking^a^	0.08	−0.03 to 0.20	0.06	1.43	0.153
Social benefits^a^	1.24	1.00 to 1.48	0.12	10.28	**< 0.001**
(Social benefits)^2^ ^a^	−0.28	−0.40 to −0.17	0.06	−4.76	**< 0.001**
(Social benefits)^3^ ^a^	−0.30	−0.41 to −0.19	0.06	−5.50	**< 0.001**
Gender [male]^b^	0.20	−0.01 to 0.40	0.10	1.90	0.057
Age^a^	−0.23	−0.33 to −0.13	0.05	−4.43	**< 0.001**
Observations	1631				
*R* ^2^/*R* ^2^ adjusted	0.147/0.143				

Predictors	Work permit				
	Estimate	CI	SE	*z*	*p*
(Intercept)	11.35	11.16 to 11.53	0.09	120.62	**< 0.001**
Empathic concern^a^	0.06	−0.05 to 0.17	0.06	1.03	0.305
Perspective‐taking^a^	0.10	−0.01 to 0.21	0.06	1.70	0.089
Work permit^a^	0.63	0.48 to 0.78	0.08	8.24	**< 0.001**
(Work permit)^2^ ^a^	−0.71	−0.91 to −0.52	0.10	−7.14	**< 0.001**
(Work permit)^3^ ^a^	−0.23	−0.31 to −0.15	0.04	−5.87	**< 0.001**
Gender [male]^b^	0.13	−0.06 to 0.33	0.10	1.36	0.174
Age^a^	−0.18	−0.28 to −0.09	0.05	−3.72	**< 0.001**
Observations	1631				
*R* ^2^/*R* ^2^ adjusted	0.137/0.133				

*Note:* Coefficients of a linear regression of empathy concern, perspective‐taking, and the personal attitude on the issue (linear, squared, cubed) on tolerance toward opinion diversity regarding refugee admission (top panel), access to social benefits (middle panel), and grating work permits (bottom panel), with gender and age as control variables. Estimates are standardized coefficients.

^a^ *z*‐transformed.

^b^ Female as reference category.

**FIGURE 3:**
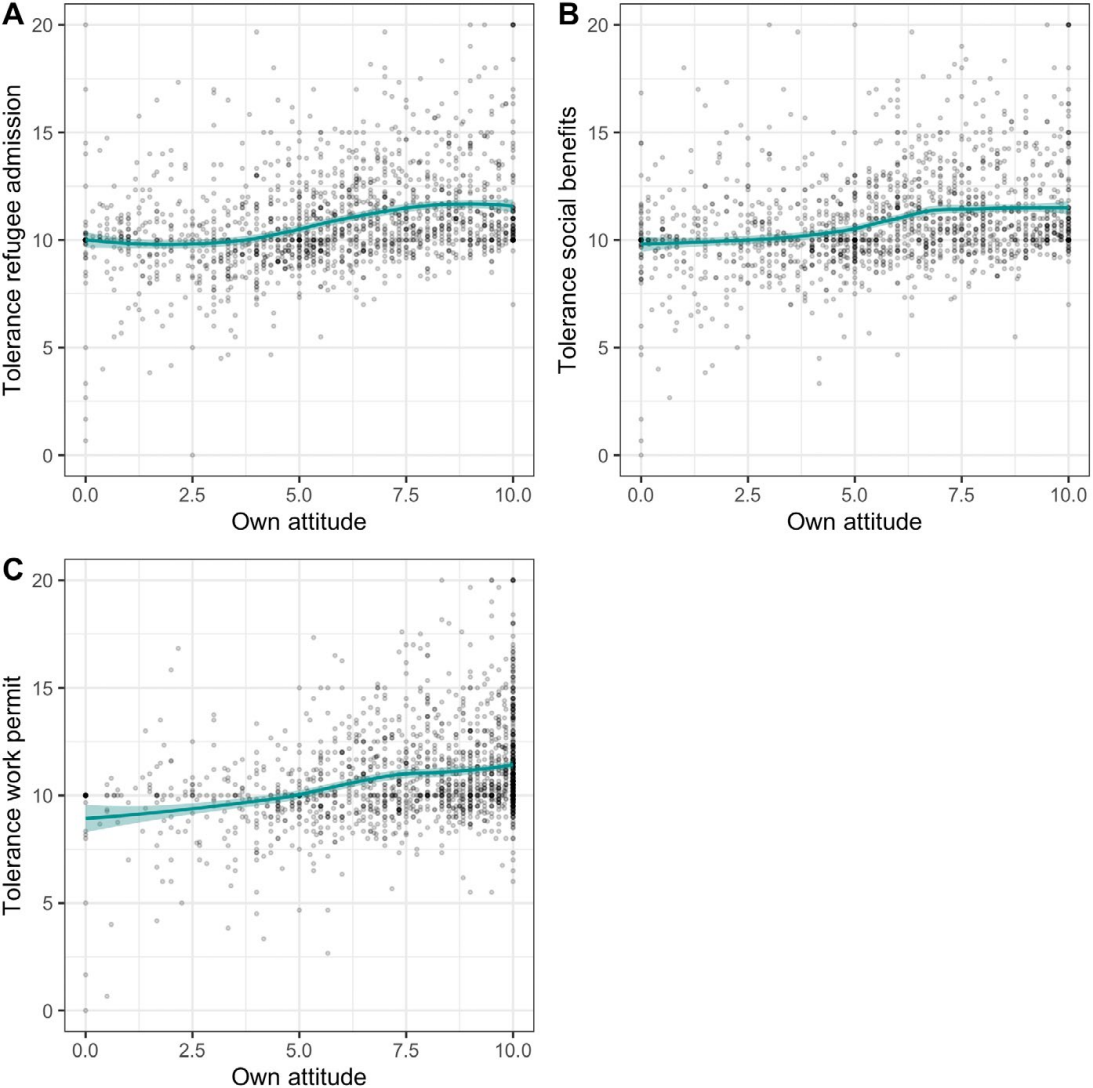
H2b: Relationship between own attitude and tolerance toward opinion diversity as a cubic function for the three policies. Regression lines for participants' own opinions on tolerance toward opinion diversity regarding (A) refugee admission, (B) social benefits, and (C) work permits. Results indicate that moderate to extreme attitudes toward pro‐refugee policies lead to more tolerance in all three items.

Our results concerning H2c are complex. Stronger support of pro‐refugee policies was positively associated with more tolerance toward opinion diversity (Table 4). However, this was not a linear relationship. Those who expressed negative attitudes toward pro‐refugee policies showed lower tolerance than those with positive attitudes and even those who were neutral or undecided; however, the level of ‘opposition’ to the policies did not matter (Figure 3).

**TABLE 4:** H3b: The relationship of empathy as well as its interactions with nationality and gender of the “other” and interpersonal closeness.

Predictors	Perceived interpersonal closeness				
	Estimates	CI	SE	*z*	*p*
(Intercept)	4.47	4.36 to 4.59	0.06	75.57	< 0.001
Empathy^a^	0.25	0.17 to 0.34	0.04	6.06	< 0.001
Nationality “other” [Syrian]^b^	−1.41	−1.48 to −1.35	0.03	−40.54	< 0.001
Nationality “other” [Ukrainian]^b^	−0.98	−1.05 to −0.92	0.03	−28.21	< 0.001
Gender “other” [male]^b^	−0.46	−0.51 to −0.40	0.03	−16.07	< 0.001
Age	0.08	0.01 to 0.15	0.04	2.19	0.029
Gender participant [male]^b^	0.36	0.21 to 0.50	0.07	4.81	0.001
Empathy^a^ × nationality Syrian	0.28	0.21 to 0.35	0.03	8.09	< 0.001
Empathy^a^ × nationality Ukrainian	0.25	0.18 to 0.32	0.03	7.24	< 0.001
Observations	7194				
Marginal *R* ^2^/conditional *R* ^2^	0.182/0.571				

*Note:* The relationship of empathic concern and perspective‐taking as well as the nationality of the other person with perceived interpersonal closeness was analyzed using a linear mixed model with empathy and nationality of the other person as well as their interaction as fixed effects and subject as random effect. The gender of the other person, gender of the participant, and age of the participant were added as control variables. Estimates are standardized coefficients.

^a^ *z*‐transformed.

^b^ German and female as reference category.

### 3.3 Empathy and Interpersonal Closeness Toward Others, Including Outgroup Members (RQ3)

Inspecting the univariate distribution, our data suggests that participants showed a medium level of interpersonal closeness toward others (*M =* 3.64, SD = 1.84). Pairwise comparisons show that participants felt closest to their ingroup (Germans, *M =* 4.45, SD = 1.75), then Ukrainians (*M =* 3.45, SD = 1.73), and then Syrians (*M =* 3.02, SD = 1.76; all items with a theoretical range = 1–7, all *p*s < 0.001).

Supporting H3a, higher empathy was associated with feeling closer to others in general, irrespective of their nationality (*β* = 0.43, *p* < 0.001, CI [0.36–0.50]; full model in Table S4).

Supporting H3b, members of the national ingroup (Germans) were perceived as being closer compared to members of a national outgroup (Ukrainians, Syrians; Table 3).

**FIGURE 4:**
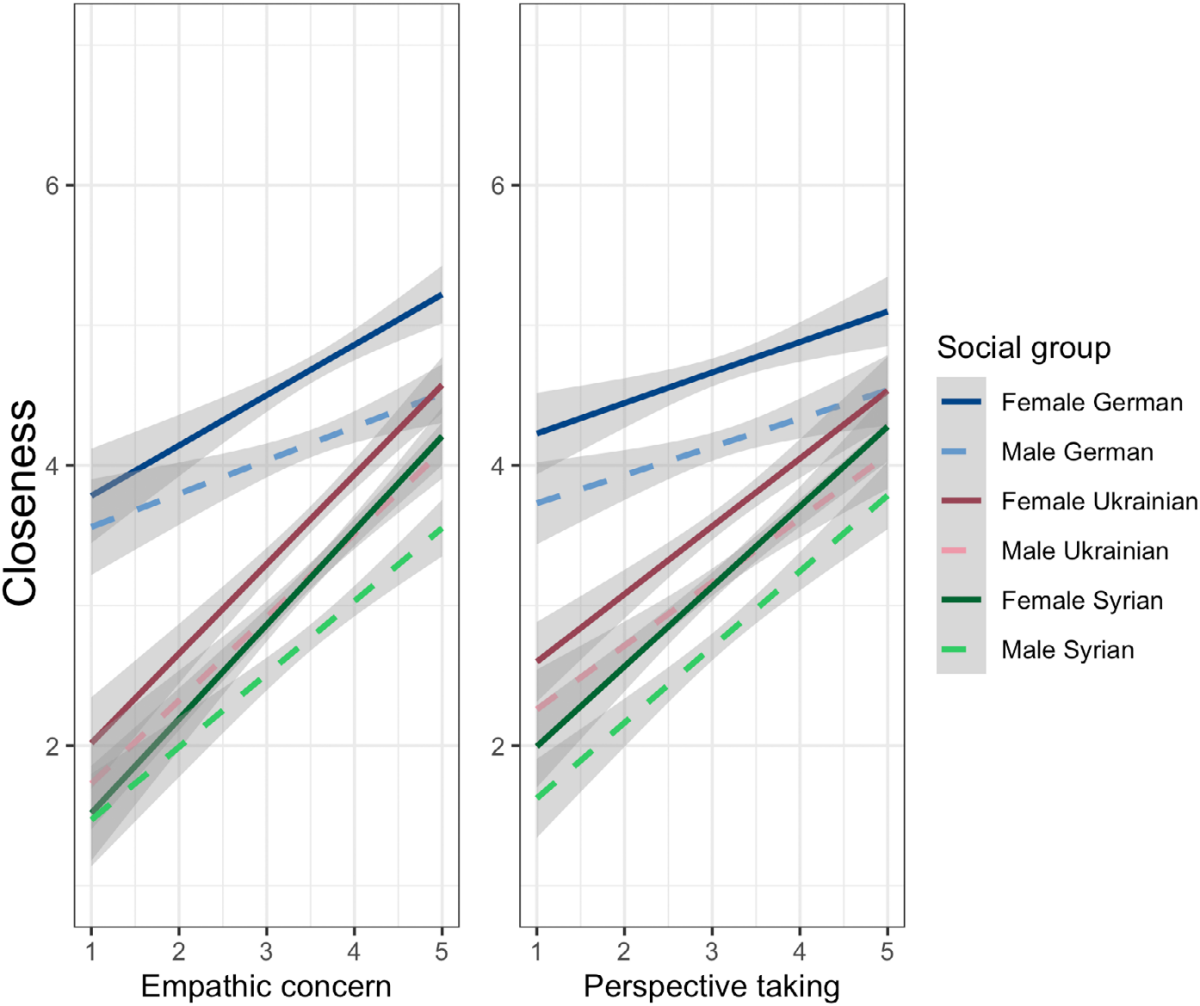
H3c: Interpersonal closeness for different social group members, separately for empathic concern and perspective‐taking. The figure shows the regression lines for empathic concern (left) and perspective‐taking (right) on interpersonal closeness for gender and nationality of the other person. Gray‐shaded areas indicate 95% confidence intervals.

Supporting H3c, higher empathy was associated with more closeness to members of the outgroup (Table 3; Figure 4). In other words, more empathetic participants not only feel closer to others in general but also differentiate less between members of in‐ and outgroups.

We found no clear support for H3d. Higher empathic concern and higher perspective‐taking were both related to higher interpersonal closeness (Figure 4, full model in Table S5).

## 4 Discussion

The study contributes to our understanding of the role of empathy in times of societal crisis—here in the context of refugee movements at the beginning of Russia's war on Ukraine.^3^ Our data showed that empathy is systematically related to solidarity and social cohesion, namely in the form of support of pro‐refugee policies, a higher tolerance for opinion diversity, and perceived interpersonal closeness to others, including social outgroups.

### 4.1 Empathy and Attitudes Toward Pro‐Refugee Policies

Regarding the first research question, the results confirm our hypothesis that higher levels of empathy are associated with more favorable attitudes toward pro‐refugee policies, namely the admission of refugees, their access to social benefits, and the granting of work permits. As predicted, these relationships seem to be primarily based on the emotional facet of empathy, empathetic concern. This finding aligns with broader theories of empathy (e.g., Batson 1990; De Waal 2008), indicating that individuals with higher emotional empathy are more likely to demonstrate sympathy and compassion toward those in need. The findings also align with recent research that found that humanitarianism is connected with support for immigration and that empathy moderates the effect of media information inducing humanitarian concern and corresponding policy preferences (Newman et al. 2015; Schieferdecker 2023). Empathy might therefore be of central importance to foster humanitarianism toward refugees.

Future research should follow up on our findings by testing whether empathy can also translate into more pro‐refugee political engagement (e.g., participating in demonstrations) and helping behavior (e.g., donating money or offering housing). In doing so, future studies may combine the links between empathy, prosocial dispositions, and helping behaviors in a comprehensive model to test the causal chain of effects.

### 4.2 Trait Empathy and Tolerance for Opinion Diversity

The second part of our study examines how higher empathy is related to more tolerance regarding differing opinions on refugee policies. In line with previous research (Butrus and Witenberg 2013; Hu and Lee 2018), we find that more empathetic individuals showed more tolerance toward people forming diverse opinions. Again, confirming the findings of previous work, we find that more empathic individuals can integrate potentially conflicting viewpoints (Eisenberg et al. 2006). However, for the individual refugee policies as well as on a facet level of empathy, the effects only remained statistically significant for perspective‐taking on tolerance toward refugee admission. One explanation for this could be that respondents' empathy with refugees conflicted with the understanding of opinions that are against pro‐refugee policies. However, the effects of participants' own opinions on tolerance suggest a different conclusion: The significant cubic effect of one's own attitude on tolerance toward opinion diversity indicates that individuals who showed support were more tolerant toward diverse opinions, regardless of their strength of opinion. Therefore, a pro‐refugee attitude also goes in line with more tolerance toward opinions that include an opposing stance on refugees.

This finding is worth spending more time with since it is not fully in line with previous research: First, previous research had suggested that a moderate opinion goes hand in hand with more tolerance toward opposing opinions (see, e.g., Ganzach and Schul 2021; Toner et al. 2013) and less polarization in the context of the immigration of refugees (Herold et al. 2023). Quite contrary, in our study, a generally pro‐refugee attitude led participants to be more tolerant toward other opinions, regardless of their opinion strength.

Second, one might assume that balancing empathy for refugees and an understanding of those who are against pro‐refugee policies might evoke an inner conflict. However, our results suggest that especially those who are in favor of admitting refugees, granting them social benefits, and permitting them to work also show the most understanding for those against these rights. Our study suggests that more empathetic individuals possess an ability to impartially evaluate diverse and intricate viewpoints, even when those viewpoints are at odds with their own attitudes.

To sum up, the results of the second research question indicate a positive link between empathy and tolerance toward differing opinions in the context of refugee movements. This questions the notion that a moderate opinion always fosters understanding whereas a more extreme opinion hinders tolerance.

### 4.3 Empathy and Interpersonal Closeness

The third research question focused on the role of trait empathy for ingroup–outgroup dynamics, specifically interpersonal closeness to Germans and two refugee groups. The results demonstrate that empathy can be positively related to perceptions of closeness: individuals who exhibited higher levels of empathy perceived others as overall closer. Additionally, the nationality of the other person impacted the closeness ratings: German participants felt closest to Germans, followed by Ukrainians and Syrians. Importantly, more empathetic individuals felt less difference in their closeness to these national groups, indicating that empathy can mitigate ingroup–outgroup categorizations concerning nationality.

Our results showed that categorization based on personal characteristics, such as nationality, plays a significant role in shaping perceptions of ingroup and outgroup members within society. A number of interrelated factors may contribute to the varying treatment of Syrian and Ukrainian refugees. First, Ukraine had just been invaded at the time of data collection, whereas the war in Syria started over a decade ago. Moreover, Ukraine is geographically much closer to Germany than Syria. These factors may result in less psychological distance and may have made the suffering more relatable (De Coninck 2022; see also Trope and Liberman 2010). In addition, the selective solidarity for Ukrainian refugees may happen due to ‘othering’ processes that are rooted in racialized and racist stereotypes: Research has shown that refugees from ethnicities that are perceived to be more similar in attitudes and values with members of the receiving country are higher valued by the domestic population (Bansak, Hainmueller, and Hangartner 2016; Esses, Hamilton, and Gaucher 2017; see also Paré 2022). On the opposite side, non‐white and non‐European refugees are often associated with cultural and economic threats to the national ingroup in public opinion and media discourses (e.g., Berning and Schlueter 2016; De Coninck 2022; Meidert and Rapp 2019). Having said all of this, it is important to finally note that higher empathic concern and perspective‐taking not just mitigated ingroup–outgroup differences but also the differences between Ukrainians and Syrians. This supports earlier work that demonstrated that other‐oriented empathy predicted higher social closeness and warmer feelings toward Syrian refugees (Pawlicka, Kazmierczak, and Jagiello‐Rusilowski 2019).

### 4.4 Applied Implications

Our study provides robust evidence that empathy is related to the way society reacts to people seeking refuge in a crisis. Higher levels of trait empathy in the population could help overcome group categorization based on racial stereotypes and could potentially facilitate smoother integration, social support, and acceptance of refugees in host societies. Previous research has demonstrated that trait empathy can be successfully trained in interventions (meta‐analytical findings in Teding van Berkhout and Malouff 2016). Interventions that have been carried out in hostile ethnic conflicts (e.g., Bruneau and Saxe 2012; Malhotra and Liyanage 2005; Stürmer et al. 2006; Todd, Bodenhausen, and Galinsky 2012) could also be applied to the context of newly arrived refugees in host countries. Empathy training could also be integrated into intergroup contact interventions to increase the effectiveness of personal social contact between refugees and citizens in the receiving countries (see also Knappert et al. 2021).

### 4.5 Limitations

While the findings of our study provide valuable insights, several limitations should be acknowledged. First, our data deals with refugees and refugee‐related policies of a specific war from the perspective of citizens of a specific country. The role of empathy in response to refugee movements outside a war context may differ. Second, we only measured how much understanding people showed toward different opinions on refugee‐related policies. Future studies could narrow the focus to understanding political *opponents* and use broader measures of group‐related emotions, e.g., from the literature on affective polarization (see, e.g., Herold et al. 2023). Third, we only explained the gender and nationality of the “other” in our IOS scales. This leaves much to the imagination of the respondents. The different refugee groups may be systematically associated with other characteristics like a certain age or education. Future research could investigate additional demographic factors that may influence interpersonal ingroup–outgroup categorization in the migration context. Fourth, and relatedly, parts of our sample may not identify as being part of a White, Christian, non‐migrant, German ingroup. In future studies, subjective measures of group belonging and identification should be added. Fifth, we did not consider personality traits and wider political ideology as conditions of the relationship between empathy and other‐related variables. For instance, Hudson and Uneal (2023) found that personality and ideology are closely connected to empathetic and counter‐empathetic (i.e., schadenfreude) responses. Finally, due to the ad‐hoc nature of the data collection, we did not preregister the analysis. As such, our findings should be considered as one data point and call for replications.

## 5 Conclusions

Our study contributes to the understanding of the relationship between empathy and political attitudes, tolerance, and ingroup–outgroup dynamics in the German population during the Russian war in Ukraine. We could show that parts of the German public reacted in prosocial ways to the flight of refugees despite the uncertainties, threats, and domestic conflicts that the war meant for the German population during the initial months. Particularly, people with higher trait empathy showed more positive attitudes toward refugees, more tolerance toward diverse perspectives, and less ingroup–outgroup categorizations. Promoting empathy and challenging stereotypes may facilitate social cohesion, intergroup relations, and the integration of refugees into host societies. Further research should explore interventions aimed at promoting empathy and examine the role of intergroup contact in improving empathy, intergroup relations, and acceptance of refugees, as well as the role of other personality traits and political ideology.

## Author Contributions

R.D. conceptualization, data curation, formal analysis, investigation, project administration, methodology, validation, writing – original draft preparation, writing – review and editing. L.M. conceptualization, data curation, methodology, project administration, writing – review and editing. D.S. conceptualization, funding acquisition, methodology, writing – original draft preparation, writing – review and editing. H.S. funding acquisition, methodology, supervision, writing – review and editing. S.H. funding acquisition, supervision, writing – review and editing. J.S. conceptualization, funding acquisition, methodology, supervision, writing – review and editing.

## Conflicts of Interest

The authors declare no conflicts of interest.

## Supporting information


Table S1.
Table S2.
Table S3.
Table S4.
Table S5.

## Data Availability

Data and code to reproduce all analyses are available at DOI: 10.17605/OSF.IO/E5XUZ.

## Endnotes

^1^
The multi‐disciplinary panel survey was conducted as an ad‐hoc study to monitor potential shifts in public opinion in the very first days of the Russian invasion. Since an expedited ethical review process was not available at the authors' institutions, the authors consulted with colleagues across various involved departments. Collectively, we concluded that the planned study posed minimal risks to participants for three reasons. First, the design was purely observational. Second, the questionnaires did not expose participants to any new information that they were not already encountering in their daily lives at the time of the data collection. Third, the survey's topic was fully disclosed from the onset. Given the timeliness and social and theoretical relevance of the study, we initiated the first survey waves before receiving the ethics committee's decision. Our internal assessment was subsequently supported by the formal review (which did not raise any ethical concern and granted the formal clearance before wave 5).

^2^
The panel provider did not offer a non‐binary choice for the quote stratification.

^3^
We want to explicitly distance ourselves from the use of the term “refugee crisis” as it can imply that a crisis is predominantly or even solely caused by those seeking refuge.
