# Supplementary material for: Empathy During Crises: Investigating Attitudes, Tolerance, and Ingroup–Outgroup Dynamics in Response to Refugee Movements
Source: J Pers. 2025 Jan 27;93(6):1351–66. doi: 10.1111/jopy.13012 (PMC12592582; doi:10.1111/jopy.13012)
Supplement: Supplementary file 1 — Table S1. Table S2. Table S3. Table S4. Table S5. [file JOPY-93-1351-s001.docx]

**Online Supplement**

| **Table S1**  *Robustness analysis for H2b and H2c excluding the values above the leverage threshold for the influential cases* | | | | | |
| --- | --- | --- | --- | --- | --- |
|  | **Refugee admission** | | | | |
| *Predictors* | *Estimate* | *CI* | *SE* | *z* | *p* |
| (Intercept) | 10.78 | 10.61 – 10.96 | 0.09 | 119.62 | **<.001** |
| Empathic concern^(1)^ | 0.07 | -0.06 – 0.20 | 0.07 | 1.04 | .297 |
| Perspective-taking^(1)^ | 0.13 | 0.01 – 0.25 | 0.06 | 2.04 | **.041** |
| Attitude admission^(1)^ | 1.10 | 0.85 – 1.34 | 0.13 | 8.69 | **<.001** |
| (Attitude admission)^2(1)^ | -0.06 | -0.18 – 0.06 | 0.06 | -0.94 | .349 |
| (Attitude admission)^3(1)^ | -0.22 | -0.34 – -0.10 | 0.06 | -3.70 | **<.001** |
| Gender [male] ^(2)^ | 0.05 | -0.16 – 0.26 | 0.11 | 0.47 | .636 |
| Age^(1)^ | -0.19 | -0.29 – -0.08 | 0.05 | -3.48 | **.001** |
| Observations | 1586 | | | | |
| R^2^ / R^2^ adjusted | 0.120 / 0.116 | | | | |
|  | **Social benefits** | | | | |
| *Predictors* | *Estimate* | *CI* | *SE* | *z* | *p* |
| (Intercept) | 10.95 | 10.78 – 11.12 | 0.09 | 125.66 | **<.001** |
| Empathic concern^(1)^ | 0.09 | -0.04 – 0.22 | 0.06 | 1.41 | .159 |
| Perspective-taking^(1)^ | 0.11 | -0.01 – 0.23 | 0.06 | 1.76 | .079 |
| Social benefits ^(1)^ | 1.20 | 0.97 – 1.44 | 0.12 | 9.92 | **<.001** |
| (Social benefits)^2(1)^ | -0.27 | -0.39 – -0.15 | 0.06 | -4.49 | **<.001** |
| (Social benefits)^3(1)^ | -0.30 | -0.41 – -0.19 | 0.06 | -5.24 | **<.001** |
| Gender [male] ^(2)^ | 0.19 | -0.02 – 0.39 | 0.10 | 1.80 | .071 |
| Age^(1)^ | -0.22 | -0.32 – -0.12 | 0.05 | -4.30 | **<.001** |
| Observations | 1585 | | | | |
| R^2^ / R^2^ adjusted | 0.142 / 0.138 | | | | |
|  | **Work permit** | | | | |
| *Predictors* | *Estimate* | *CI* | *SE* | *z* | *p* |
| (Intercept) | 11.42 | 11.23 – 11.60 | 0.09 | 121.45 | **<.001** |
| Empathic concern^(1)^ | 0.10 | -0.02 – 0.22 | 0.06 | 1.68 | .093 |
| Perspective-taking^(1)^ | 0.07 | -0.05 – 0.18 | 0.06 | 1.16 | .248 |
| Work permit^(1)^ | 0.92 | 0.73 – 1.11 | 0.10 | 9.45 | **<.001** |
| (Work permit)^2(1)^ | -0.97 | -1.19 – -0.75 | 0.11 | -8.69 | **<.001** |
| (Work permit)^3(1)^ | -0.47 | -0.59 – -0.35 | 0.06 | -7.48 | **<.001** |
| Gender [male] ^(2)^ | 0.16 | -0.03 – 0.35 | 0.10 | 1.64 | .101 |
| Age^(1)^ | -0.18 | -0.27 – -0.08 | 0.05 | -3.68 | **<.001** |
| Observations | 1583 | | | | |
| R^2^ / R^2^ adjusted | 0.121 / 0.117 | | | | |
| *Note.* ^(1)^ z-transformed ^(2)^Dummy coded with female as reference category | | | | | |

| **Table S2**  *The relationship of empathy and attitudes towards pro-refugee attitudes (H1a)* | | | | | |
| --- | --- | --- | --- | --- | --- |
|  | **Attitude towards refugees** | | | | |
| *Predictors* | *Estimates* | *CI* | *std. Error* | *z value* | *p* |
| (Intercept) | 6.01 | 5.85 – 6.18 | 0.08 | 72.66 | **<.001** |
| Empathy^(1)^ | 0.70 | 0.58 – 0.81 | 0.06 | 12.00 | **<.001** |
| Gender [male]^(2)^ | 0.65 | 0.41 – 0.88 | 0.12 | 5.49 | **<.001** |
| Age^(1)^ | 0.03 | -0.09 – 0.14 | 0.06 | 0.44 | .662 |
| Observations | 1,631 | | | | |
| R^2^ / R^2^ adjusted | 0.089 / 0.087 | | | | |
| *Note.* ^(1)^ z-transformed, ^(2)^ Dummy coded with female as reference category | | | | | |

| **Table S3**  *The relationship of empathy with tolerance towards diverse opinions (H2a)* | | | | | |
| --- | --- | --- | --- | --- | --- |
|  | **Tolerance different opinions** | | | | |
| *Predictors* | *Estimates* | *CI* | *std. Error* | *z value* | *p* |
| (Intercept) | 32.21 | 31.80 – 32.61 | 0.21 | 156.06 | **<.001** |
| Empathy^(1)^ | 0.95 | 0.66 – 1.23 | 0.15 | 6.52 | **<.001** |
| Gender [male]^(2)^ | -0.62 | -0.90 – -0.33 | 0.14 | -4.26 | **<.001** |
| Age^(3)^ | 0.87 | 0.29 – 1.45 | 0.29 | 2.97 | **.003** |
| Observations | 1631 | | | | |
| R^2^ / R^2^ adjusted | 0.037 / 0.035 | | | | |
| *Note.* ^(1)^ z-transformed, *Mean* (*SD*) of the original value were 3.39 (0.67), ^(2)^ Dummy coded with female as reference category, ^(3)^z-transformed, *Mean* (*SD*) of the original values were 47.09 (13.87) | | | | | |

| **Table S4**  *The relationship of empathy and perceived interpersonal closeness (H3a)* | | | | | |
| --- | --- | --- | --- | --- | --- |
|  | **Perceived interpersonal closeness** | | | | |
| *Predictors* | *Estimates* | *CI* | *std. Error* | *z value* | *p* |
| (Intercept) | 3.44 | 3.34 – 3.55 | 0.05 | 64.01 | **<.001** |
| Empathy^(1)^ | 0.43 | 0.36 – 0.50 | 0.04 | 11.79 | **<.001** |
| Gender [male]^(2)^ | 0.08 | 0.01 – 0.15 | 0.04 | 2.19 | **.029** |
| Age^(3)^ | 0.36 | 0.21 – 0.50 | 0.07 | 4.81 | **<.001** |
| Observations | 7194 | | | | |
| R^2^ / R^2^ adjusted | 0.059 / 0.423 | | | | |
| *Note.* ^(1)^ z-transformed, *Mean* (*SD*) of the original value were 3.39 (0.67), ^(2)^ Dummy coded with female as reference category, ^(3)^z-transformed, *Mean* (*SD*) of the original values were 47.09 (13.87) | | | | | |

| **Table S5**  *The relationship of empathic concern and perspective taking and perceived interpersonal closeness as well as their interaction with nationality and gender of the “other” (H3c)* | | | | | |
| --- | --- | --- | --- | --- | --- |
|  | **Interpersonal closeness** | | | | |
| *Predictors* | *Estimates* | *CI* | *SE* | *z* | *p* |
| (Intercept) | 4.46 | 4.35 – 4.58 | 0.06 | 71.23 | **<.001** |
| Empathic concern (EC)^(1)^ | 0.23 | 0.13 – 0.32 | 0.05 | 4.52 | **<.001** |
| Perspective-taking (PT)^(2)^ | 0.07 | -0.03 – 0.16 | 0.05 | 1.33 | .185 |
| Nationality [syr]^(3)^ | -1.41 | -1.48 – -1.35 | 0.03 | -40.55 | **<.001** |
| Nationality [ukra]^(3)^ | -0.98 | -1.05 – -0.92 | 0.03 | -28.22 | **<.001** |
| Gender other [m]^(4)^ | -0.46 | -0.51 – -0.40 | 0.03 | -16.07 | **<.001** |
| Age participant | 0.07 | -0.01 – 0.14 | 0.04 | 1.81 | .070 |
| Gender participant [male] ^(4)^ | 0.38 | 0.23 – 0.52 | 0.07 | 5.05 | **<.001** |
| EC * nationality [syr] | 0.12 | 0.04 – 0.20 | 0.04 | 2.85 | **.004** |
| EC * nationality [ukra] | 0.19 | 0.11 – 0.27 | 0.04 | 4.58 | **<.001** |
| PT * nationality [syr] | 0.20 | 0.12 – 0.29 | 0.04 | 4.95 | **<.001** |
| PT * nationality [ukra] | 0.10 | 0.02 – 0.18 | 0.04 | 2.40 | **.017** |
| Observations | 7194 | | | | |
| Marginal R^2^ / Conditional R^2^ | 0.184 / 0.571 | | | | |
| *Note.* ^(1)^ z-transformed, *Mean* (*SD*) of the original values were 3.56 (0.76), ^(2)^ z-transformed, *Mean* (*SD*) of the original values were 3.18 (0.77), ^(3)^ Nationality was dummy coded with Germans as reference category, ^(4)^ Gender of the other person and gender of participant was dummy coded with females as reference category | | | | | |
